# Impact of SNPs, off-targets, and passive permeability on efficacy of BCL6 degrading drugs assigned by virtual screening and 3D-QSAR approach

**DOI:** 10.1038/s41598-022-25587-3

**Published:** 2022-12-06

**Authors:** Solmaz Karimi, Farzaneh Shahabi, Shaden M. H. Mubarak, Hanie Arjmandi, Zahra Sadat Hashemi, Navid Pourzardosht, Alireza Zakeri, Mahdieh Mahboobi, Abolfazl Jahangiri, Mohammad Reza Rahbar, Saeed Khalili

**Affiliations:** 1 Laboratory of Mitochondrial Biology and Metabolism, Nencki Institute of Experimental Biology of Polish Academy of Sciences, 02-093 Warsaw, Poland; 2 Faculty of Advanced Technologies in Medical Sciences, Golestan University of Medical Sciences, Gorgan, Iran; 3 Department of Clinical Laboratory Science, Faculty of Pharmacy, University of Kufa, Najaf, Iraq; 4 Faculty of Pharmacy, Islamic Azad University of Amol Branch, Amol, Iran; 5 ATMP Department, Breast Cancer Research Center, Motamed Cancer Institute, ACECR, Tehran, Iran; 6 Biochemistry Department, Guilan University of Medical Sciences, Rasht, Iran; 7 Department of Biology Sciences, Shahid Rajaee Teacher Training University, Tehran, Iran; 8 Applied Microbiology Research Center, Systems Biology and Poisonings Institute, Baqiyatallah University of Medical Sciences, Tehran, Iran; 9 Pharmaceutical Sciences Research Center, Shiraz University of Medical Sciences, Shiraz, Iran

**Keywords:** Lung cancer, Cancer therapy, Drug development

## Abstract

B-cell lymphoma 6 (BCL6) regulates various genes and is reported to be overexpressed in lymphomas and other malignancies. Thus, BCL6 inhibition or its tagging for degradation would be an amenable therapeutic approach. A library of 2500 approved drugs was employed to find BCL6 inhibitory molecules via virtual screening. Moreover, the 3D core structure of 170 BCL6 inhibitors was used to build a 3D QSAR model and predict the biological activity. The SNP database was analyzed to study the impact on the destabilization of BCL6/drug interactions. Structural similarity search and molecular docking analyses were used to assess the interaction between possible off-targets and BCL6 inhibitors. The tendency of drugs for passive membrane permeability was also analyzed. Lifitegrast (DB11611) had favorable binding properties and biological activity compared to the BI-3802. Missense SNPs were located at the essential interaction sites of the BCL6. Structural similarity search resulted in five BTB-domain containing off-target proteins. BI-3802 and Lifitegrast had similar chemical behavior and binding properties against off-target candidates. More interestingly, the binding affinity of BI-3802 (against off-targets) was higher than Lifitegrast. Energetically, Lifitegrast was less favorable for passive membrane permeability. The interaction between BCL6 and BI-3802 is more prone to SNP-derived variations. On the other hand, higher nonspecific binding of BI-3802 to off-target proteins could bring about higher undesirable properties. It should also be noted that energetically less desirable passive membrane translocation of Lifitegrast would demand drug delivery vehicles. However, further empirical evaluation of Lifitegrast would unveil its true potential.

## Introduction

B-cell lymphoma 6 (BCL6) is a transcriptional repressor and a member of the BTB/POZ-zinc finger family. This protein is described as a promising drug target for non-Hodgkin lymphomas^1,2^. BCL6 plays a crucial role in normal immunity and is required in the germinal center response for high-affinity antibody production^3,4^. On the other hand, BCL6 is not expressed in the cells, which are required to produce high-affinity antibodies and B-cell proliferation, such as plasma cells, naive B-cells, and CD4 + T-cell subset follicular helper (Tfh) T-cells^5^. Elevated BCL6 expression is a common driver of B-cell malignancies. Mutations in regulatory pathways, reciprocal somatic BCL6 translocation, promoter mutation, and exonic mutation are among the provokers of BCL6 expression^6^. Diffuse large B-cell lymphomas (DLBCL) are the most common subtype of non-Hodgkin lymphoma and about 50% of DLBCL cases are associated with BLC6 expression. BCL6 regulates hundreds of DNA damage sensing^7^, cell proliferation^8^, anti-apoptosis^9^, and senescence^10^ genes to drive the malignant phenotype in DLBCL. BCL6 expression is also reported in angioimmunoblastic T-cell lymphoma, follicular lymphoma, and Burkitt lymphoma^11,12^. Moreover, BCL6 expression is reported in other malignancies. Thus, it could potentially be contemplated as a drug target in other diseases such as breast cancer^13,14^ and non-small cell lung cancer^15^.

Promising antitumor effects have recently been reported by selective targeting of BCL6. Various inhibitors have been introduced to perturb the protein–protein interaction between the BCL6 corepressor and BTB domain of BCL6. Small molecule inhibitors^1,2,16–19^, macrocycles^19,20^, and high-affinity peptides^21^ have already been developed for BCL6 inhibition. However, they are effective at high concentrations. This property limits their application as clinical therapeutic agents. Therefore, screens for more effective BCL6 inhibitors are still ongoing. The search for BCL6 inhibitors have led to identification of small molecules such as BI-3802^18^, which can induce the BCL6 degradation. They interact with the BTB domain of the BCL6, which is responsible for homo-dimerization and interaction with corepressor proteins^22^. The results of treatment with BI-3802 are comparable with the results of genetic BCL6 knockout^23^. The efficacy of these inhibitors is more pronounced than the results of non-degrading BCL6 inhibitors and heterobifunctional BCL6 degraders^18,24^.

Selective targeting of BCL6 is endowed with great potential in the fight against different diseases, especially non-Hodgkin lymphomas. Finding novel drugs, which are capable of mimicking the most successful BCL6 inhibitors (particularly degrading small molecules) would bring about higher efficiency. Bioinformatics has already been proven an amenable approach to solve various biological issues^25–28^. In the present study, we aimed to employ a novel approach to screen a library of previously approved small molecule drugs to find drugs with similar properties to BCL6 degrading small-molecules. Using a combinatorial approach virtual screening results were joined with 3DQSAR predictions to arrive at the best BCL6-inhibiting drug candidates.

## Results

### Structures and sequences

The sequence of the BCL6 protein was obtained from the Uniprot database under the ID of P41182. BCL6 is 706 amino acids in length with various experimentally resolved structures. Most of the resolved BCL6 structures are limited to its Tramtrack and Bric-à-Brac (BTB) domain in complex with other compounds. The structures under the ID of 5MW2 and 6XMX were reported to be bound to BI-3802. The SDF file format was stored for the found BCL6 inhibitors using the links provided by Binding Database. These inhibitors were cross-checked with the structures introduced in the PDB database. The resulting file contained 170 BCL6 inhibiting compounds with determined IC50 (nm) values. Although the IC50 values were available for the inhibitors, the Ki values were missing for most compounds. More than 2500 approved compounds with diverse properties were also stored in the SDF file downloaded from DrugBank. The compounds of this library have been approved for official commercialization in at least one jurisdiction at a given time. Virtual screening of this library could lead to the introduction of suitable drugs, which could be repurposed for BCL6 degradation.

### Virtual screening

**Figure 1:**
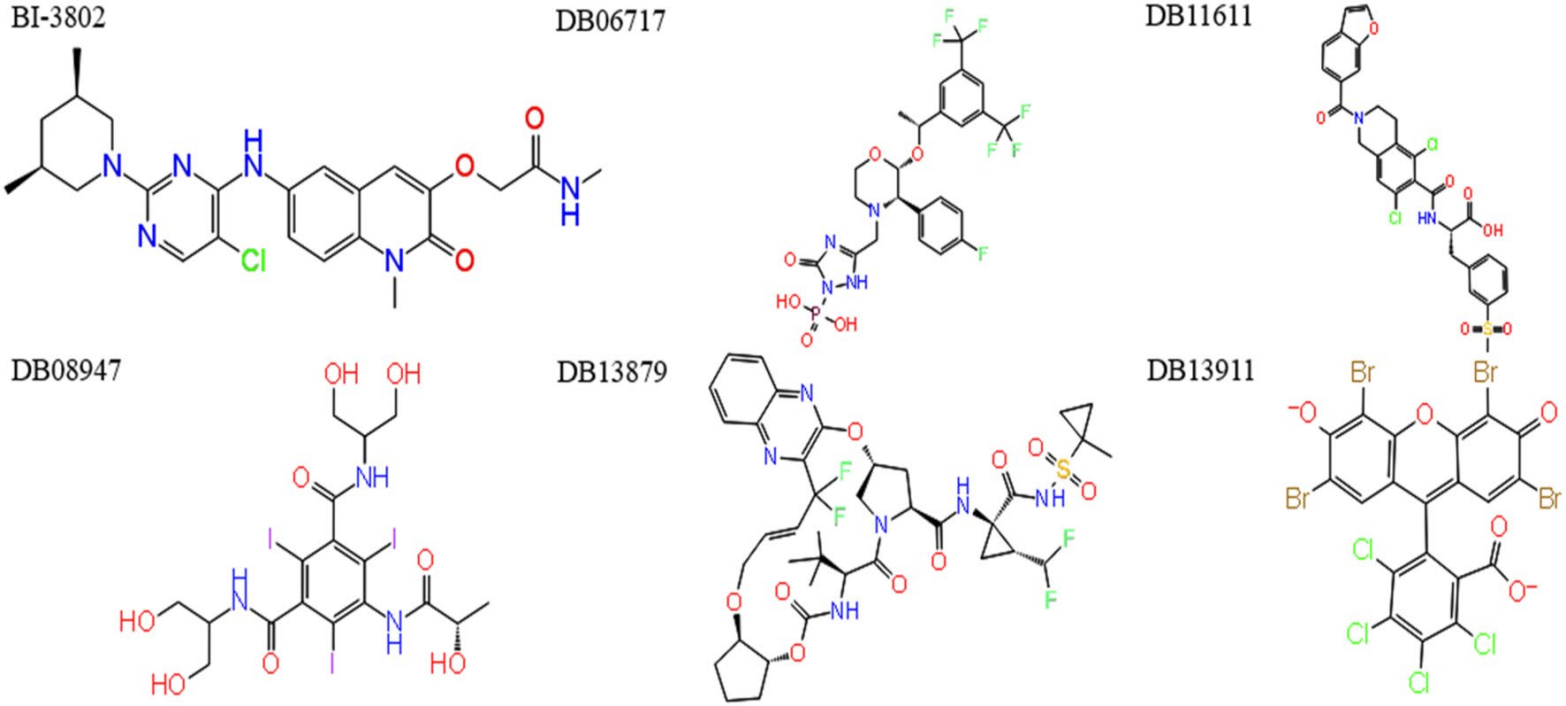
The 2D structure of BI-3802 and the top five results of virtual screening analyses with the highest binding energies.

The structure of molecules from the filtered drug library was docked against the crystalized structure of the BCL6. The final drug library contained 1967 molecules, and 542 molecules were filtered out. The generation of 3D structures increased the number of conformations for each ligand, which led to a final library with 9835 structures. Performing the virtual screening workflow, all structures were docked and assigned with a binding affinity. The top 5 molecules with the lowest binding energy were the molecules under the DrugBank ID of DB06717 (Fosaprepitant), DB11611 (Lifitegrast), DB13879 (Glecaprevir), DB13911 (Phloxine B), and DB08947 (Iopamidol). These molecules were selected for further analysis. The structures of these molecules and BI-3802 are depicted in Fig. 1. The binding energies of the BI-3802, DB06717 (Fosaprepitant), DB11611 (Lifitegrast), DB13879 (Glecaprevir), DB13911 (Phloxine B), and DB08947 (Iopamidol) were calculated to respectively be − 7.3, − 8.6, − 8.9, − 8.6, − 8.6, and − 9.2 kcal/mole against the BCL6 structure. The interactions between the top five ligands and the BCL6 protein are shown in Supplementary File 1.

### Ligand preparation to build 3D QSAR model

The similarity searches resulted in 40 inhibitors with the highest similarity to the BI-3802. This number of ligands would be enough to build a 3D QSAR model. The minimization run was set to avoid duplication of the selected ligands, which could lead to unreliable 3D QSAR models. Ligand alignment has shown that the selected inhibitors share a similar backbone. Since 3D QSAR models are built based on the experimentally determined activity values, the IC50 values were converted to PIC50 values to be used as activity factors. The SDF file of the inhibitors was edited to contain a new column representing these values.

### 3D QSAR model

The structures of the minimized and aligned inhibitors were used to build the 3D QSAR model. During the process of finding the best 3D QSAR model, six structures had the lowest performance in the prediction of activity. These structures were omitted from the data set, and the final 3D QSAR model was constructed based on 36 structures (Table 1). The maximum number of partial least squares factors was set to three and the training set was set to include 70% of the dataset. Implementing the CoMFA/CoMSIA methods, the Field-based QSAR tool managed to build the models. Various models were built with different statistical properties and checked for their quality. All Gaussian fields including the Gaussian steric, electrostatic, hydrophobic, H bond acceptor, H bond donor, and aromatic ring fields were calculated for each model.

**Table 1:** The structures employed for the 3D QSAR modeling.

No	BindingDB ligand name	IC50 (nM)	pIC50	2D structure	No	BindingDB ligand name	IC50 (nM)	pIC50	2D structure
1	CHEMBL4547183	760	6.11919		19	CHEMBL4644523	368	6.43415	
2	CHEMBL4459012	640	6.19382		20	CHEMBL4524719	230	6.63827	
3	CHEMBL4445645	520	6.284		21	CHEMBL4475438	930	6.03152	
4	CHEMBL4573672	200	6.69897		22	CHEMBL4538610	470	6.3279	
5	CHEMBL4466680	390	6.40894		23	CHEMBL4513450	1850	5.73283	
6	CHEMBL4439574	330	6.48149		24	CHEMBL4531272	1200	5.92082	
7	CHEMBL4465387	130	6.88606		25	CHEMBL4078747	5000	5.30103	
8	CHEMBL4525209	710	6.14874		26	CHEMBL4646073	152	6.81816	
9	CHEMBL4476234	150	6.82391		27	CHEMBL4063061	6100	5.21467	
10	CHEMBL4559074	86	7.0655		28	CHEMBL4096773	501	6.30016	
11	CHEMBL4458261	190	6.72125		29	CHEMBL4102754	3200	5.49485	
12	CHEMBL4560573	3500	5.45593		30	CHEMBL4483413	680	6.16749	
13	CHEMBL4574515	97	7.01323		31	CHEMBL4448503	25	7.60206	
14	CHEMBL4482923	400	6.39794		32	CHEMBL4094351	2.9	8.5376	
15	CHEMBL4463841	270	6.56864		33	CHEMBL4090962	4100	5.38722	
16	CHEMBL4469177	1000	6		34	CHEMBL4093418	6310	5.19997	
17	CHEMBL4457011	960	6.01773		35	CHEMBL4101172	3981	5.40001	
18	CHEMBL4587209	310	6.50864		36	CHEMBL4547183	760	6.1	

### Model validation

**Figure 2:**
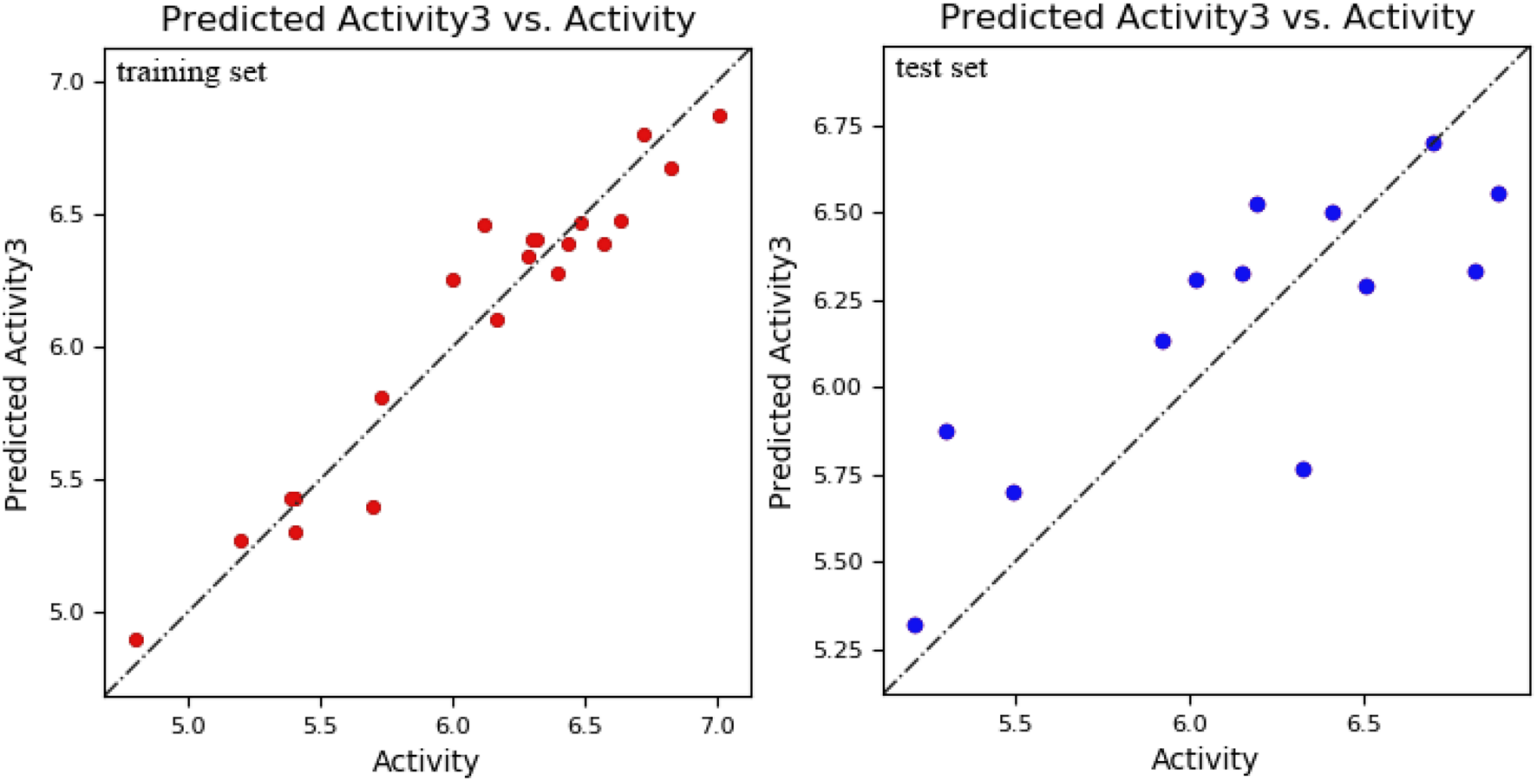
The scatter plots of actual vs predicted pIC50 values of the structures employed for 3D QSAR model development. On the left is the scatter plot for the training set structures and on the right is the scatter plot for test set structures.

**Figure 3:**
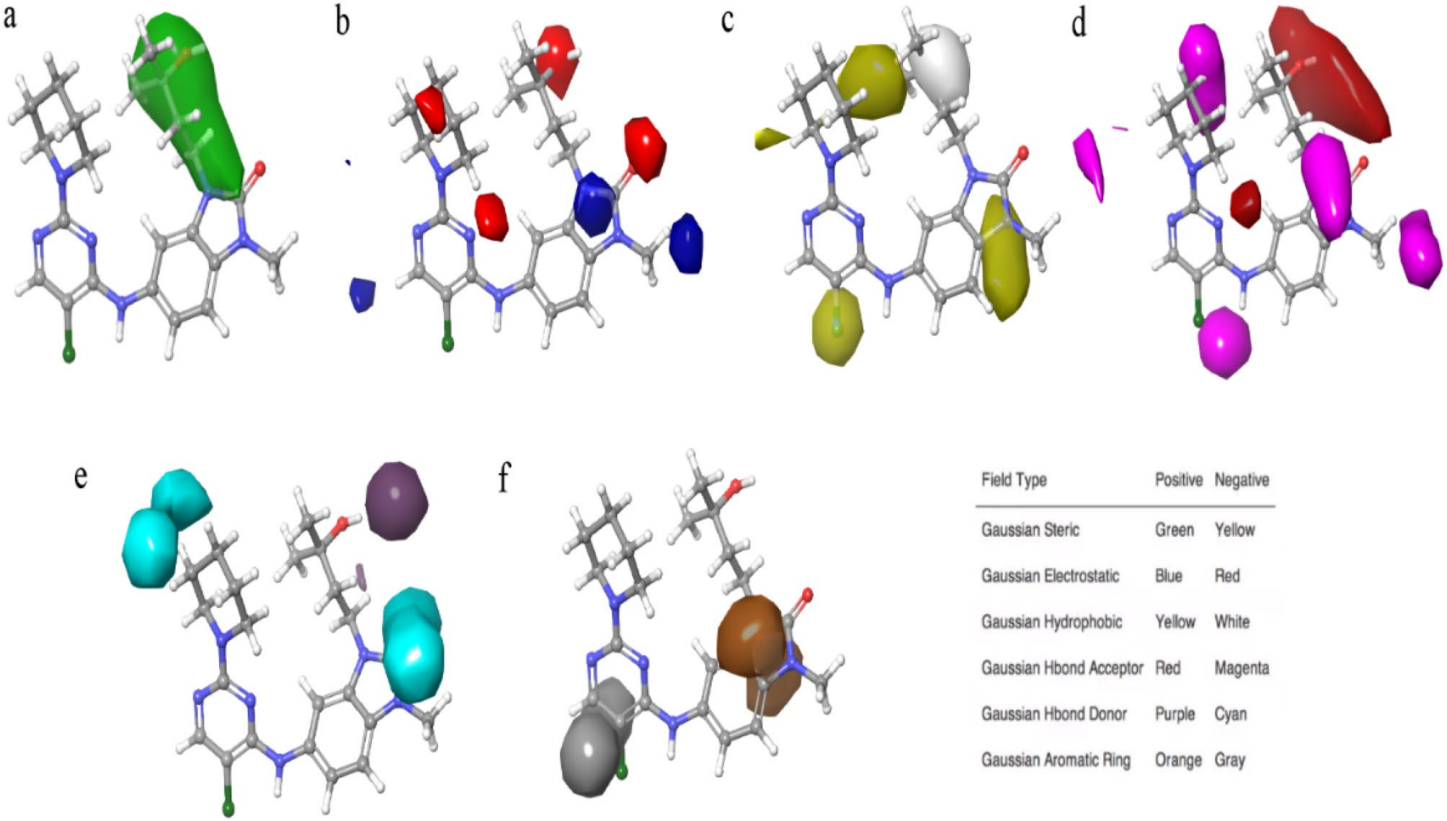
The contour representation of Gaussian steric (**a**), electrostatic (**b**), hydrophobic (**c**), H bond acceptor (**d**), H bond donor (**e**), and aromatic ring (**f**) field fractions are depicted by their intensities. One of the structures of the dataset is illustrated to make the contours more comprehensible.

The model is acceptable when it has an R^2^ value greater than 0.6, higher stability, lower RMSE value, and smaller P value^29^. Among the generated models, a model had statistically significant values. This model had R^2^ value equal to 0.93, stability value equal to 0.92, RMSE of 0.32, and P value of 2.75e−010. These PLS statistical parameters of the developed model exhibit good internal predictive power. This model was used to predict the biological activity of experimental ligands. The difference between actual activity and predicted activity is shown in Fig. 2. Moreover, the contours representing the field fractions of the Gaussian field are depicted by their intensities in Fig. 3.

### Prediction of activity

The results of the activity prediction were generated for all 3D QSAR models. The prediction of activity for BI-3802 has resulted in an activity value of 6.5. The same prediction was made for the top five hits of virtual screening. The activity values were predicted to be 6.2, 6.1, 5.9, 5.8, and 5.6 for DB06717, DB11611, DB13879, DB13911, and DB08947, respectively.

### Protein and ligand interactions

**Figure 4:**
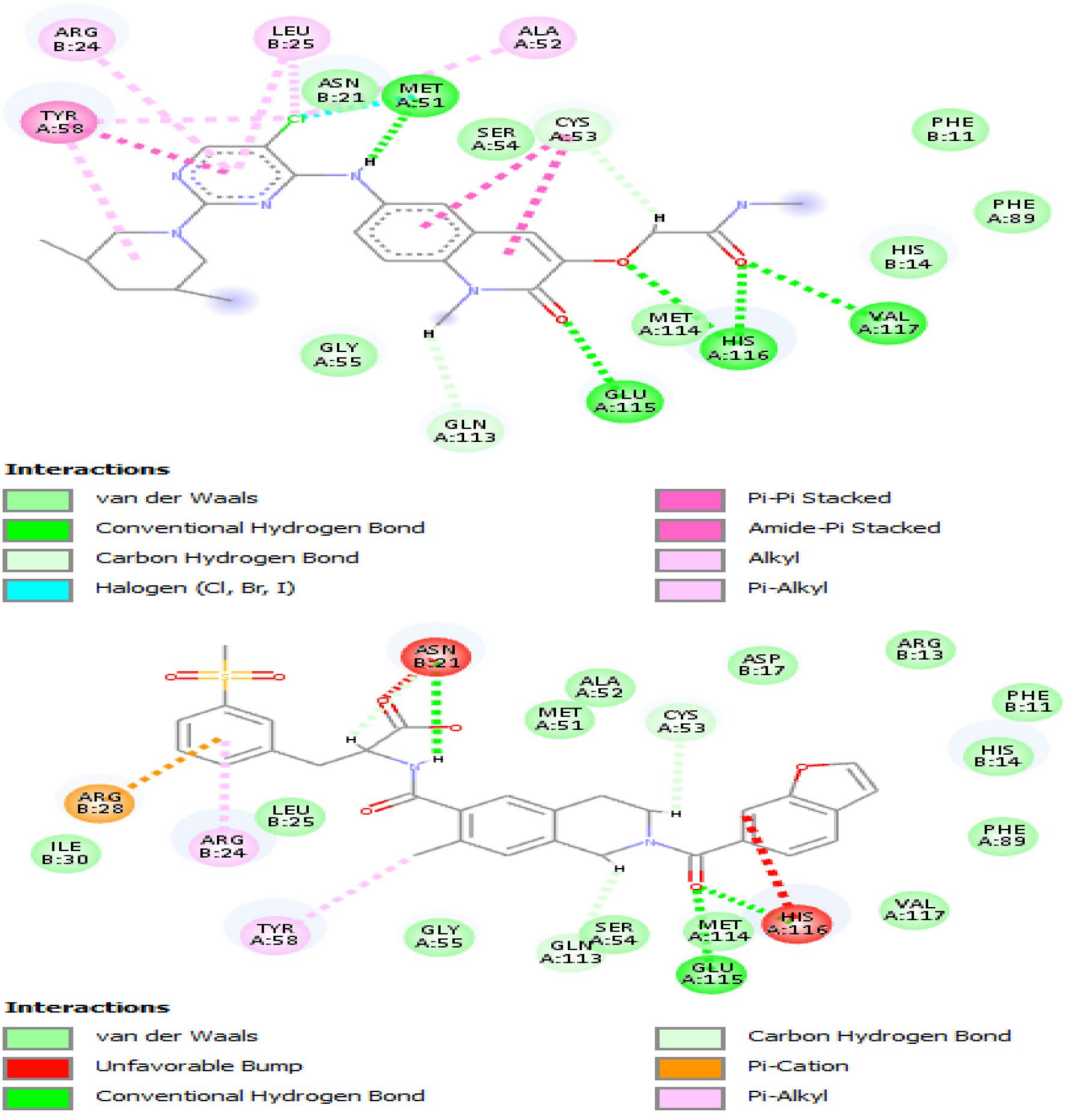
The 2D plot for interaction between BI-3802 (upper plot) and Lifitegrast (lower plot) and the BCL6. The involved amino acids are in spheres (three-letter amino acid name, chain ID, and the amino acid number in the protein sequence) and the formed bonds are depicted as a dashed line.

**Figure 5:**
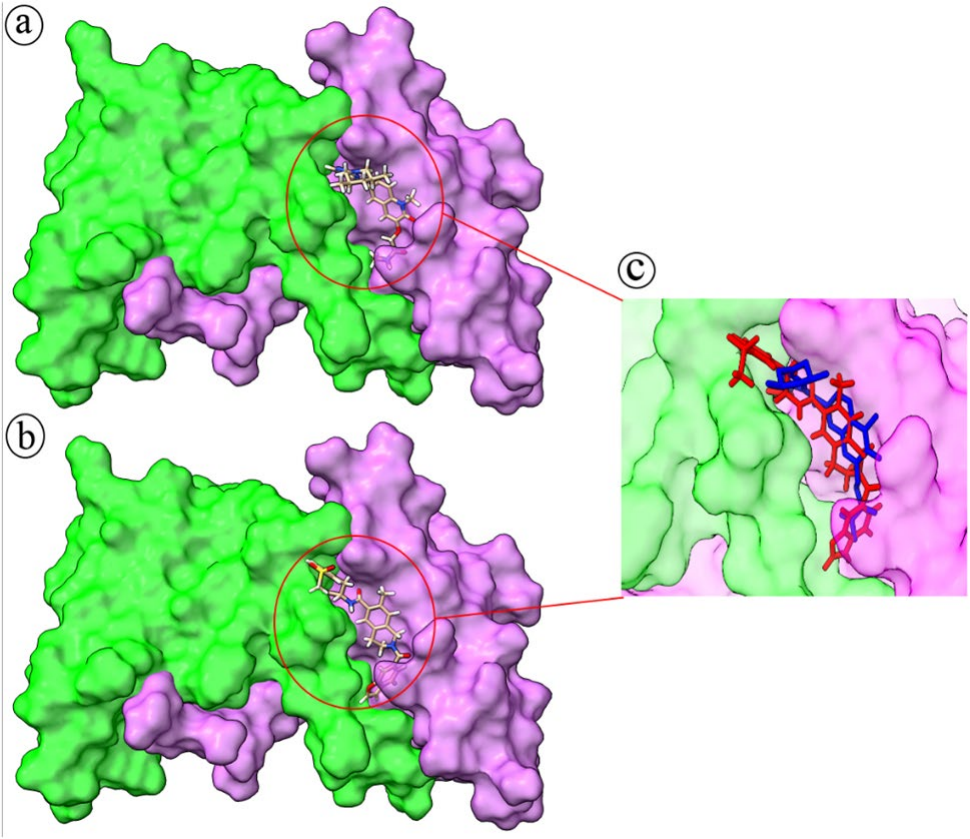
The interaction orientation for BI-3802 (**a**) and Lifitegrast (**b**) with the BCL6 protein. Chain A of the BLC6 is green and chain B is violet. Simultaneous occupation of the interaction site (**c**) by BI-3802 (blue) and Lifitegrast (red) shows their similar orientation within this site.

The discovery studio software drew the interaction between the BCL6 protein and the drug molecules. The obtained results showed that most of the involved amino acids are shared between the BI-3802 and the drug molecules. This means that the orientation of interaction between BCL6 and the selected drug molecules resembles the interaction between BCL6 and BI-3802. However, among the analyzed interactions, the interaction between the BCL6 and Lifitegrast had the highest resemblance to the BI-3802 interactions (Fig. 4). The interaction between the BCL6 and Lifitegrast also has the highest resemblance to the orientation of interaction between BI-3802 and BCL6 (Fig. 5).

### The effect of population-wide SNP variations on BCL6 interactions

Search for SNPs of BCL6 has resulted in 10,965 variations throughout its gene sequence. The variation was associated with different functional classes, including the inframe deletion, inframe indel, inframe insertion, missense, noncoding transcript variant, and synonymous. Since our study was interested in variations with amino acid changes, the search was limited to missense functional class. There were 515 variations in the missense class with one or two amino acid changes in each position (Supplementary File 2). There are 17 BCL6 amino acids involved in BCL6 and BI-380 interaction, and four extra BCL6 amino acids (a total of 21) are involved in BCL6 and Lifitegrast interactions. Among these amino acids, seven positions were subjected to missense variations. All seven amino acids were among the shared interacting amino acids of BCL6 (Table 2). The Allele Frequency Aggregator (ALFA) provides the allele frequency for each position of variation within different populations. The allele frequency data were extracted for seven BCL6 positions with amino acid variations (Table 3). None of the variations was reported in the ClinVar database for their clinical significance. It seems that SNPs of BCL6 could have significant consequences in the populations with high frequencies of these variations. This effect would be more evident in the SNPs, which are located in the sites of interaction between BCL6 and its inhibitors. Changes in these locations could results in structural changes and loss of key bonds between the BCL6 and the inhibitor.

**Table 2:** Single amino acid polymorphisms (SAP) among BLC6 amino acids, which interact with BI-3802 and DB11611.

No	BCL6/ BI-3802 Interacting amino acids	SAP	BCL6/ DB11611 Interacting amino acids	SAP
1	PHE 11	SER	PHE 11	SER
2	–	–	ARG13	–
3	HIS 14	–	HIS 14	–
4	–	–	ASP17	–
5	ASN 21	–	ASN 21	–
6	ARG 24	CYS, HIS	ARG 24	CYS, HIS
7	LEU 25	–	LEU 25	–
8	–	–	ARG28	–
9	–	–	ILE30	–
10	MET 51	–	MET 51	–
11	ALA 52	–	ALA 52	–
12	CYS 53	–	CYS 53	–
13	SER 54	–	SER 54	–
14	GLY 55	–	GLY 55	–
15	TYR 58	–	TYR 58	–
16	PHE 89	LEU	PHE 89	LEU
17	GLN 113	LYS, HIS	GLN 113	LYS, HIS
18	MET 114	–	MET 114	–
19	GLU 115	GLN	GLU 115	GLN
20	HIS 116	ARG	HIS 116	ARG
21	VAL 117	PHE	VAL 117	PHE

**Table 3:** Allele frequency for the SNPs of BLC6 gene at positions that interact with BI-3802 and DB11611.

	Study	Population	Group	Sample size	Ref allele	Alt allele
ARG24CYS	ExAC	Global	Study-wide	121,412	G = 0.999992	A = 0.000008
	ExAC	Europe	Sub	73,354	G = 1.00000	A = 0.00000
	ExAC	Asian	Sub	25,166	G = 0.99996	A = 0.00004
	ExAC	American	Sub	11,578	G = 1.00000	A = 0.00000
	ExAC	African	Sub	10,406	G = 1.00000	A = 0.00000
	ExAC	Other	Sub	908	G = 1.000	A = 0.000
	gnomAD–Exomes	Global	Study-wide	251,472	G = 0.999996	A = 0.000004
	gnomAD–Exomes	European	Sub	135,398	G = 1.000000	A = 0.000000
	gnomAD–Exomes	Asian	Sub	49,010	G = 0.99998	A = 0.00002
	gnomAD–Exomes	American	Sub	34,590	G = 1.00000	A = 0.00000
	gnomAD–Exomes	African	Sub	16,256	G = 1.00000	A = 0.00000
	gnomAD–Exomes	Ashkenazi Jewish	Sub	10,078	G = 1.00000	A = 0.00000
ARG24HIS	The avon longitudinal study of parents and children	PARENT AND CHILD COHORT	Study-wide	3854	C = 0.9997	T = 0.0003
	UK 10 K study–Twins	TWIN COHORT	Study-wide	3708	C = 1.0000	T = 0.0000
PHE89LEU	–	Total	Global	14,050	A = 1.00000	G = 0.00000
		European	Sub	9690	A = 1.0000	G = 0.0000
		African	Sub	2898	A = 1.0000	G = 0.0000
		African Others	Sub	114	A = 1.000	G = 0.000
		African American	Sub	2784	A = 1.0000	G = 0.0000
		Asian	Sub	112	A = 1.000	G = 0.000
		East Asian	Sub	86	A = 1.00	G = 0.00
		Other Asian	Sub	26	A = 1.00	G = 0.00
		Latin American 1	Sub	146	A = 1.000	G = 0.000
		Latin American 2	Sub	610	A = 1.000	G = 0.000
		South Asian	Sub	98	A = 1.00	G = 0.00
		Other	Sub	496	A = 1.000	G = 0.000
GLN113LYS	ExAC	Global	Study-wide	121,220	G = 0.999992	T = 0.000008
	ExAC	Europe	Sub	73,234	G = 0.99999	T = 0.00001
	ExAC	Asian	Sub	25,146	G = 1.00000	T = 0.00000
	ExAC	American	Sub	11,560	G = 1.00000	T = 0.00000
	ExAC	African	Sub	10,376	G = 1.00000	T = 0.00000
	ExAC	Other	Sub	904	G = 1.000	T = 0.000
	gnomAD–Exomes	Global	Study-wide	251,340	G = 0.999996	T = 0.000004
	gnomAD–Exomes	European	Sub	135,266	G = 0.999993	T = 0.000007
	gnomAD–Exomes	Asian	Sub	49,008	G = 1.00000	T = 0.00000
	gnomAD–Exomes	American	Sub	34,592	G = 1.00000	T = 0.00000
	gnomAD–Exomes	African	Sub	16,256	G = 1.00000	T = 0.00000
	gnomAD–Exomes	Ashkenazi Jewish	Sub	10,078	G = 1.00000	T = 0.00000
	gnomAD–Exomes	Other	Sub	6140	G = 1.0000	T = 0.0000
GLN113HIS	–	Total	Global	10,680	C = 1.00000	G = 0.00000
		European	Sub	6962	C = 1.0000	G = 0.0000
		African	Sub	2294	C = 1.0000	G = 0.0000
		African Others	Sub	84	C = 1.00	G = 0.00
		African American	Sub	2210	C = 1.0000	G = 0.0000
		Asian	Sub	108	C = 1.000	G = 0.000
		East Asian	Sub	84	C = 1.00	G = 0.00
		Other Asian	Sub	24	C = 1.00	G = 0.00
		Latin American 1	Sub	146	C = 1.000	G = 0.000
		Latin American 2	Sub	610	C = 1.000	G = 0.000
		South Asian	Sub	94	C = 1.00	G = 0.00
		Other	Sub	466	C = 1.000	G = 0.000
GLU115GLN	–	Total	Global	10,680	C = 1.00000	G = 0.00000
		European	Sub	6962	C = 1.0000	G = 0.0000
		African	Sub	2294	C = 1.0000	G = 0.0000
		African Others	Sub	84	C = 1.00	G = 0.00
		African American	Sub	2210	C = 1.0000	G = 0.0000
		Asian	Sub	108	C = 1.000	G = 0.000
		East Asian	Sub	84	C = 1.00	G = 0.00
		Other Asian	Sub	24	C = 1.00	G = 0.00
		Latin American 1	Sub	146	C = 1.000	G = 0.000
		Latin American 2	Sub	610	C = 1.000	G = 0.000
		South Asian	Sub	94	C = 1.00	G = 0.00
		Other	Sub	466	C = 1.000	G = 0.000
HIS116ARG	gnomAD–Exomes	Global	Study-wide	251,318	T = 0.999996	C = 0.000004
	gnomAD–Exomes	European	Sub	135,252	T = 1.000000	C = 0.000000
	gnomAD–Exomes	Asian	Sub	49,006	T = 1.00000	C = 0.00000
	gnomAD–Exomes	American	Sub	34,590	T = 0.99997	C = 0.00003
	gnomAD–Exomes	African	Sub	16,254	T = 1.00000	C = 0.00000
	gnomAD–Exomes	Ashkenazi Jewish	Sub	10,078	T = 1.00000	C = 0.00000
	gnomAD–Exomes	Other	Sub	6138	T = 1.0000	C = 0.0000
VAL117PHE	–	Total	Global	660	C = 1.000	A = 0.000
		European	Sub	78	C = 1.00	A = 0.00
		African	Sub	434	C = 1.000	A = 0.000
		African Others	Sub	0	C = 0	A = 0
		African American	Sub	434	C = 1.000	A = 0.000
		Asian	Sub	34	C = 1.00	A = 0.00
		East Asian	Sub	34	C = 1.00	A = 0.00
		Other Asian	Sub	0	C = 0	A = 0
		Latin American 1	Sub	0	C = 0	A = 0
		Latin American 2	Sub	0	C = 0	A = 0
		South Asian	Sub	6	C = 1.0	A = 0.0
		Other	Sub	108	C = 1.000	A = 0.000

### Off-target screening and analyses

Performing nine iterations of PSI-BLAST, there were no new sequences added to the BLAST results. Therefore, the PSI-BLAST iterations were stopped at nine. As expected, most of the results belong to the BCL6 BTB domain, and there are some other similar structures, which contain TB domains. The top five sequences with the highest query coverage and identity were selected as possible off-targets of the BI-3802 and DB11611. Table 4 indicates the data for these sequences. The top five similar structures to the BCL6 BTB domain were docked with both BI-3802 and Lifitegrast structures. The docking results indicated that BI-3802 and Lifitegrast could bind the selected structures with a high binding affinity (Table 5). More interestingly, the binding affinity of BI-3802 was lower than Lifitegrast against four out of five selected similar structures. Only in the case of the structure under the PDB ID of 3OHU, the binding affinity was lower for the complex made by BI-3802. This means that BI-3802 makes more stable complexes with off-target proteins with similar sequences to the BTB domain of BCL6.

**Table 4:** The results of top five similar structures to BCL6 BTB domain.

	Protein Name	Query coverage (%)	Identity	PDB ID
1	Zinc finger and BTB domain of LRF	96	31.36%	2IF5
2	Transcription regulator protein BACH2	95	41.03	3OHU
3	Transcription regulator protein BACH1	97	36.16%	2IHC
4	MIZ1-BTB-domain	95	35.34%	7AZW
5	BTB domain of HKR3	95	32.48%	3B84

**Table 5:** The binding affinity between BI-3802 and Lifitegrast and top five similar structures to BCL6 BTB domain. The binding affinity of eight docking iteration for each complex are listed.

Ligand	Binding affinity kcal/mol	Ligand	Binding affinity kcal/mol
2IF5-BI-3802	− 8.4	2IF5-Lifitegrast	− 8.3
2IF5-BI-3802	− 7.2	2IF5-Lifitegrast	− 7.8
2IF5-BI-3802	− 7	2IF5-Lifitegrast	− 7.8
2IF5-BI-3802	− 7	2IF5-Lifitegrast	− 7.8
2IF5-BI-3802	− 7	2IF5-Lifitegrast	− 7.7
2IF5-BI-3802	− 6.9	2IF5-Lifitegrast	− 7.6
2IF5-BI-3802	− 6.9	2IF5-Lifitegrast	− 7.5
2IF5-BI-3802	− 6.8	2IF5-Lifitegrast	− 7.5
2IF5-BI-3802	− 6.8	2IF5-Lifitegrast	− 7.4
2IHC-BI-3802	− 8.6	2IHC-Lifitegrast	− 8.2
2IHC-BI-3802	− 8.2	2IHC-Lifitegrast	− 7.8
2IHC-BI-3802	− 7.8	2IHC-Lifitegrast	− 7.7
2IHC-BI-3802	− 7.4	2IHC-Lifitegrast	− 7.5
2IHC-BI-3802	− 7.4	2IHC-Lifitegrast	− 7.4
2IHC-BI-3802	− 7.3	2IHC-Lifitegrast	− 7.4
2IHC-BI-3802	− 7.3	2IHC-Lifitegrast	− 7.4
2IHC-BI-3802	− 7.1	2IHC-Lifitegrast	− 7.4
2IHC-BI-3802	− 7	2IHC-Lifitegrast	− 7.4
3B84-BI-3802	− 7.8	3B84-Lifitegrast	− 7.4
3B84-BI-3802	− 7.6	3B84-Lifitegrast	− 7.3
3B84-BI-3802	− 7.6	3B84-Lifitegrast	− 7.2
3B84-BI-3802	− 7.2	3B84-Lifitegrast	− 7.1
3B84-BI-3802	− 7.1	3B84-Lifitegrast	− 6.9
3B84-BI-3802	− 7	3B84-Lifitegrast	− 6.8
3B84-BI-3802	− 7	3B84-Lifitegrast	− 6.8
3B84-BI-3802	− 6.9	3B84-Lifitegrast	− 6.7
3B84-BI-3802	− 6.8	3B84-Lifitegrast	− 6.6
3OHU-BI-3802	− 8.2	3OHU-Lifitegrast	− 9.9
3OHU-BI-3802	− 8	3OHU-Lifitegrast	− 9.7
3OHU-BI-3802	− 8	3OHU-Lifitegrast	− 9.3
3OHU-BI-3802	− 7.9	3OHU-Lifitegrast	− 9
3OHU-BI-3802	− 7.8	3OHU-Lifitegrast	− 8.9
3OHU-BI-3802	− 7.8	3OHU-Lifitegrast	− 8.9
3OHU-BI-3802	− 7.6	3OHU-Lifitegrast	− 8.8
3OHU-BI-3802	− 7.6	3OHU-Lifitegrast	− 8.8
3OHU-BI-3802	− 7.5	3OHU-Lifitegrast	− 8.8
7AZW-BI-3802	8.7	7AZW-Lifitegrast	− 7.7
7AZW-BI-3802	− 8.4	7AZW-Lifitegrast	− 7.7
7AZW-BI-3802	− 8.3	7AZW-Lifitegrast	− 7.6
7AZW-BI-3802	− 8.2	7AZW-Lifitegrast	− 7.5
7AZW-BI-3802	− 8.1	7AZW-Lifitegrast	− 7.5
7AZW-BI-3802	− 7.8	7AZW-Lifitegrast	− 7.3
7AZW-BI-3802	− 7.7	7AZW-Lifitegrast	− 7.3
7AZW-BI-3802	− 7.7	7AZW-Lifitegrast	− 7.2
7AZW-BI-3802	− 7.7	7AZW-Lifitegrast	− 7.2

### Passive membrane permeability

**Figure 6:**
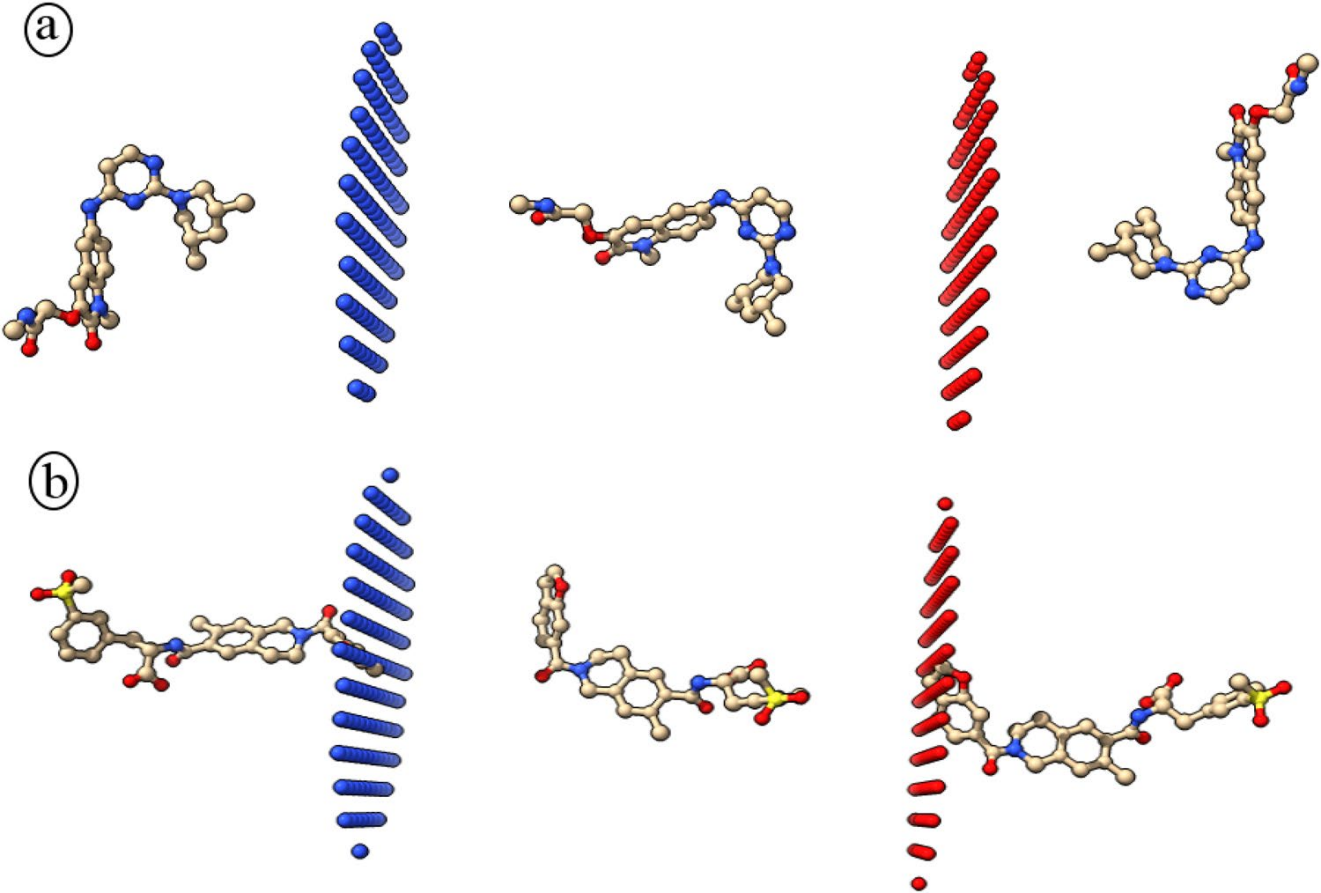
Schematic illustration of BI-3802 (**a**) and Lifitegrast (**b**) translocation through the membrane bilayer predicted by PerMM. The green and red plates are the outer and inner leaflets of the membrane.

Passive permeability of molecules through the lipid bilayer is one of the features, which should be taken in to account for drug candidates with intra cellular targets. A drug candidate with low permeability should be treated accordingly to enable passing through plasma membrane. This analysis would help to make necessary provisions for application of low permeability molecules as drug candidates. The obtained results indicated that the BI-3802 has a higher (− 3.62 kcal/mol) free energy of binding against dioleoyl-phosphatidylcholine (DOPC) bilayer in comparison to the Lifitegrast (− 4.17 kcal/mol). Moreover, the permeability coefficient of BI-3802 and Lifitegrast was calculated to be − 5.61 and − 4.49 for black lipid membranes (BLM), respectively (Fig. 6). Given the obtained permeability coefficient for blood–brain barrier (BBB), both drugs were impermeable for BBB. The free energy changes during the membrane passage showed that transmission of Lifitegrast is energetically less favorable.

## Discussion

BCL6 has already been suggested as a promising drug target for non-Hodgkin lymphomas. Elevated expression of BCL6 is a common driver of B cell malignancies. Given these circumstances, BCL6 has been subjected to the design of inhibitory molecules^30^. Recently, small molecules have been introduced that can hijack the cellular quality control machinery and induce protein degradation. BI-3802 is a small molecule that acts as a molecular glue to enforce a novel interaction between an E3 ligase and the BCL6 complex and its ultimate tagging for degradation^31^. In light of this evidence, we have devised an integrative 3D QSAR-based virtual screening on repurposing the previously approved drugs for BCL6 inhibition and degradation.

The approved drug library was evaluated for efficient BTB domain binding and similar biological activity to BI-3802. Among the screened molecules, the binding affinity of five molecules was higher than the BI-3802. Various interactions between the ligand molecules and the BCL6 protein resulted in the elevated binding affinities. All of the found molecules are among the approved molecules. Fosaprepitant is an intravenously administered antiemetic drug, Glecaprevir is an antiviral agent, Phloxine B is a polycyclic aromatic compound acting as colorant in dental disclosing tablets, Iopamidol is a contrast agent, and Lifitegrast is a competitive antagonist of lymphocyte function-associated antigen-1 (LFA-1), which is used to treat dry eye disease and is supplied as an eye drop. Visual inspection of the interaction between these drugs and the BCL6 showed that the Lifitegrast shares a similar binding orientation with the BI-3802. Moreover, the 2D interaction plot of these two molecules shared a significant number of identical amino acids. These properties could be construed as the high potential of Lifitegrast to mimic the BI-3802 functions with higher affinity and, consequently lower concentrations. This notion was also corroborated by the results of 3DQSAR prediction for the biological activity of BI-3802 and Lifitegrast. Similar biological activity prediction for these molecules further confirms the possibility of their similar effects on BCL6 targeting. Since Lifitegrast is an approved drug, it has already been assessed for its systemic side effects, efficacy, and safety^32,33^. Therefore, it could be a more amenable drug candidate for BCL6 inhibition and degradation. It should be noted that Lifitegrast is administered as an eye drop and it is not systemically introduced to the whole body. The clinical trials of this drug assess its properties, safety, and tolerability issues as an ophthalmic solution^34^. This is while the fight against lymphoma would need a systemic approach, which requires further investigations about the systemic side effects, efficacy, and safety of Lifitegrast. In this regard, the other four drugs with high binding affinity against BCL6 are more advantageous.

Lifitegrast is the generic name of the Lifitegrast small molecule drug. Lifitegrast is an FDA-approved drug for the treatment of keratoconjunctivitis sicca or dry eye syndrome (DED). It is a derivative of tetrahydroisoquinoline, which is developed through the rational design process. DED is associated with elevated levels of inflammatory cytokines, which are expressed by T-lymphocytes. The binding between LFA-1 and intercellular adhesion molecule-1 (ICAM-1) forms the immunological synapse and facilitates the proliferation/activation of T-cell, the release of cytokine, and more T-cells recruitment at the inflammatory sites^35^. Lifitegrast is shown to block the LFA-1/ICAM-1 interaction. This molecule could inhibit the release of tumor necrosis factor-alpha (TNF-α), interferon δ, cytokines, and other interleukins (ILs)^32^. Therefore, Lifitegrast acts as an anti-inflammatory agent by obstructing immunological synapses. Inflammation has also been suggested to be strongly correlated with cancer. It has been implied that inflammatory infiltration plays a pivotal role in the development of malignancies. Inflammatory cells and soluble mediators are among the micro-environmental factors, which are responsible for induced angiogenesis, sustained cell growth, and invasion, and suppressed antitumor immune functions^36^. Lymphomas develop specialized tissue microenvironments, which are composed of different cell populations. Tumor microenvironments have recently been suggested to have relevant clinical roles and emerged as novel targets for treatment. Thus, lymphoma microenvironments, which are deprived of inflammatory factors, would be more susceptible to tumor inhibition. Given the anti-inflammatory effects of Lifitegrast, it could be used for the microenvironment-directed treatment of human lymphomas. Together with possible anti-BCL6 effects, the anti-inflammatory effects of Lifitegrast would serve well for lymphoma therapy. The anti-inflammatory effects of Lifitegrast, in combination with its BCL6 inhibitory effects, promise an amenable drug candidate for lymphoma treatment.

Analyzing the SNP profile of the BCL gene, various positions were marked with missense variations. It has already been demonstrated that BCL6 variations could have functional consequences^37–39^. Among these variations, seven positions were involved in the interactions between BCL6 and both BI-3802 and DB11611. Since the SNPs at these positions could have a significant effect on the binding affinity between BCL6 and its blocking agents, they could reduce the possibility of forming a focal plane and the subsequent degradation. Although the allele frequency data showed that none of these variations is highly frequent in different populations, genetic analyses of the nonresponding individuals could shed light on the mechanism behind failed BCL6 degradation (via BI-3802 or DB11611) in populations with these SNPs. Moreover, it seems that the interaction between BCL6 and BI-3802 would be more susceptible to SNP variations. Three out of four hydrogen bond-forming amino acids of the BCL6/BI-3802 complex (GLU 115, HIS 116, and VAL 117) are the missense SNP positions, while this number is only two out of three for BCL6/Lifitegrast complex (GLU 115 and HIS 116). It means that the BCL6/BI-3802 complex is more prone to lose the highly stabilizing hydrogen bonds upon the SNP-derived variations. The higher binding affinity of the BCL6/Lifitegrast complex is mainly devoted by numerous Van Der Vaal’s interactions, which are not affected by SNP-derived variations.

Off-target binding of a small molecule of therapeutic interest is the nonspecific binding of that molecule to a protein target other than the intended primary target. It has been reported that poly-specificity is frequently (~ 25%) displayed by various bio-therapeutics. This property results in severe patient adverse events and failed clinical trials^40,41^. In an attempt to find off-target candidates for BI-3802 or Lifitegrast, we have performed a structural similarity search and docking study. The off-target candidates were all BTB-domain-containing proteins, and some were POK (POZ and Kru¨ppel) family proteins, which have already been shown to play pivotal roles in human cell development and differentiation^7,42^. The obtained results indicated that off-target binding properties of both BI-3802 and Lifitegrast are similar to BCL6 binding. This similarity in chemical behavior and binding properties of BI-3802 and Lifitegrast against off-target candidates could be construed as their potential functional similarity. In other words, due to their high chemical fitness to interact with essential amino acids of the BTB-domain, BI-3802 and Lifitegrast could share a common functional property in tagging BTB-domain-containing proteins to degradation. Moreover, these analyses showed that BI-3802 and Lifitegrast could even have a better binding affinity toward these off-targets. Since some of these proteins such as LRF (leukemia/lymphoma-related factor) have oncogenic properties^43^, the effects of BI-3802 and Lifitegrast on their degradations could be worth further analyses. More interestingly, the binding affinity analyses showed that unlike the interaction with BCL6, the binding affinity of BI-3802 (against off-targets) is lower than Lifitegrast. This means BI-3802 could make stronger bonds with the off-target proteins compared to the Lifitegrast. Higher nonspecific binding of BI-3802 could bring about side effects that are more undesirable and the need for higher in vivo dosage of the drug to get significant results. Given these circumstances, Lifitegrast seems to be a more amenable drug candidate to tag BCL6 for degradation.

Lifitegrast is an antagonist of LFA-1 (a single-pass transmembrane protein). Thus, it does not need to be translocated through the plasma membrane to exert its therapeutic effects against dry eye syndrome. Lifitegrast showed a higher binding affinity to the outer and inner surfaces of the membrane, which reduces its tendency for membrane disassociation. Since most of the found off-targets are intracellular proteins, the calculated low passive membrane permeability and the low tendency for membrane disassociation could decrease the potential side effects. In contrast, molecular tagging of BCL6 for degradation requires the translocation of drug candidates through the plasma membrane. Hence, the free energy for its translocation through the membrane is highly unfavorable. These properties are also disadvantages of Lifitegrast for passive permeation through the plasma membrane. However, BI-3802 has a lower binding affinity to the outer and inner surfaces of the membrane and energetically its translocation is more favorable. These properties of BI-3802 are evident from its ability for foci formation^31^. In this regard, various drug delivery systems could be employed for effective translocation through the plasma membrane. Site-targeting, sustained or controlled release, protection of pharmaceuticals from degradation and clearance, improved therapeutic effects, and fewer harmful side effects are some of the benefits of drug delivery systems. Various systems, including liposomes, exosomes, and nanostructures, could be exploited for Lifitegrast delivery^44–46^.

It could be concluded that Lifitegrast could act as a potential BCL6 targeting agent with possible BCL6 degrading and anti-inflammatory effects. This molecule has a comparable binding affinity and predicted biological activity as of BI-3802. Moreover, the binding affinity in BCL6/Lifitegrast complex is mostly devoted by numerous Van Der Vaal’s interactions, which are less prone to be affected by SNP-derived variations. It also should be pinpointed that a higher binding affinity of BI-3802 against possible off-target proteins could bring about undesirable side effects and the need for higher in vivo dosage of the drug to get significant results during in vivo administrations. The lower tendency of Lifitegrast for passive translocation through the plasma membrane should also be considered in the design of delivery systems for this therapeutic. However, further empirical assessments would bring about reliable insights regarding the efficacy of Lifitegrast as an alternative treatment for lymphomas. Taken together, the approach of the current study could be adapted for the design of other small molecule degrading drugs to get higher efficacy with fewer undesirable properties.

## Methods

### Structures and sequences

The sequence of the BCL6 protein was fetched from the Uniprot database (https://www.uniprot.org/). The three-dimensional (3D) structure of BCL6 in complex with different blocking agents was found in Protein Data Bank (https://www.rcsb.org/search). More information about the structure and function of the BCL6 inhibitors was searched, in the PubChem database (https://pubchem.ncbi.nlm.nih.gov/). The Binding Database (https://www.bindingdb.org/bind/index.jsp) was searched for possible inhibitors of the BCL6 protein. The BCL6 protein name was searched, using the target search tool of the Binding Database. The structure of the resulting binding molecules was stored in SDF file format. The DrugBank database (https://go.drugbank.com/) was used to download its approved library of drugs. The library was stored in SDF file format.

### Virtual screening

The virtual screening was conducted to find out which one of the molecules from the approved drug library could fit in the binding site of the BCL6 protein with high binding affinity. The crystallized structure of the BCL6 protein in the 6XMX complex was separated from its inhibitor using the ConTEXT software. The final drug library was prepared by filtering out the molecules smaller than 200 Da and higher than 1000 Da using the ligand filtration panel of the Maestro. Virtual screening of the approved drug library was performed against the structure of BCL6 using the PyRx software^47^. The approved drug library contains the 2D structure of each compound. Therefore, the 3D version of this library was produced by exploiting the LigPrep panel of the Maestro software (v 11.8.012). OPLS_2005 force fields were used to prepare the ligands with high quality and correct chirality. The AutoDock tools software was used to convert the BCL6 structure and the approved drug library into the PDBQT file format. The molecules were prepared by adding hydrogen atoms and merging all nonpolar hydrogens. The AutoDock Vina (Trott and Olson 2010) interface of the PyRex software was used to dock the compounds of the approved drug library against the crystalized structure of BCL6. The docking grid was centered on the location of the BI-3802 within the 6XMX complex. The van der Waals and electrostatic terms were calculated using the set- and distance-dependent dielectric functions of the AutoDock Vina software. Other parameters of the PyRex screening software were defined by adapting a method previously employed by Khalili et al.^48^.

### Preparation of 3D QSAR dataset

The obtained inhibitor library from the Binding Database was searched for the ligands with the highest similarity to the BI-3802. The “find similar molecules by fingerprints” tool of the Discovery Studio client software (v 16.1.0.15350) was used to find similar molecules. The minimal similarity value was set to 0.50, the similarity coefficient was set to Tanimoto, output contributions were set as True, and properties were used from FCFC_6 predefined set. The BI-3802 was set as the reference molecule. The resulting molecules were then minimized using the Micromodel minimization tool of the Maestro software. The minimized molecules were then aligned using the Flexible Ligand Alignment tool of the Maestro software. The experimentally determined IC50 values of the selected inhibitors were converted to pIC50 values using the following equation:

$${\text{PIC5}}0\, = \,{9} - {\text{Log}}_{{{1}0}} \,({\text{IC5}}0).$$

### Building a 3D QSAR model

The field-based QSAR workflow of the Maestro software was used to build the 3D QSAR model. Field-based QSAR tool implements the CoMFA/CoMSIA methods with a specific set of parameters. To build the model, this method takes the Lennard–Jones steric potentials from the OPLS_2005 force field. The atomic charges for the electrostatic fields are also taken from the OPLS_2005 force field. Hydrophobic fields are based on the atom types, and hydrophobic parameters, which are described by Ghose et al.^49^. Phase pharmacophore feature definitions were used to define hydrogen bond acceptor, hydrogen bond donor, and aromatic ring fields with projected points. The minimized and aligned dataset of the inhibitors was used as the input dataset. PIC50 values were determined as activity values for the prepared inhibitors. The training and test sets were chosen randomly, and 70% of the inhibitors were set to be the training set. The Extended Gaussian field was selected to build the model. This field uses all Gaussian fields, including the Gaussian steric, electrostatic, hydrophobic, H bond acceptor, H bond donor, and aromatic ring fields. The maximum number of partial least squares factors was set to be three and other values were left as default values. Various 3D QSAR models were built using these settings. All outlier structures were omitted to form the dataset. The percent of the training set and the maximum number of partial least squares factors were adjusted accordingly.

### Model validation

The 3D QSAR models were analyzed for their accuracy and statistical properties. An important method to assess the quality and internal predictive capacity of 3D-QSAR models is the partial least squares multivariate regression analysis. This method exploits the combination of the calculated fields and bioactivity data^50^. Internal parameters were computed by the execution of a leave-one-out cross-validation analysis. Test set compounds were used to examine the generated QSAR models. The overall significance of the model was evaluated by calculation of statistical parameters and comparison of the predicted and experimentally determined pIC50 values. The 3D QSAR models could have outstanding external predictive capacity if they met the requirements for statistical parameters. To arrive at the best 3D QSAR model, the models with a low value of R^2^ for the regression and low stability of the model predictions (upon changes in the training set composition) were discarded. The best-performed 3D QSAR model with satisfactory properties was selected as the final 3D QSAR model.

### Activity prediction

The selected 3D QSAR model was used to predict the activity for BI-3802, DB06717, DB11611, DB13879, DB13911, and DB08947. To perform the prediction, the structures of these molecules were minimized and aligned with the rest of the structures from the QSAR modeling dataset. The same tools were employed to do the minimization and alignment. The activity prediction was carried out for the molecules using the prediction option of the QSAR modeling workflow.

### Generation of 2D plot

The discovery studio software was employed to draw the 2D plots of interactions between the BCL6 structure and BI-3802, DB06717, DB11611, DB13879, DB13911, and DB08947structures. The 2D interaction plots would bring about more insights into the key amino acids involved in the interaction between the protein and the drug molecules. Moreover, different bonds formed between the BCL6 protein and the drug molecules would be unraveled. The complexes of BCL6 protein and ligands were obtained from the virtual screening results.

### Evaluation of population-wide SNP variations

The dbSNP (https://www.ncbi.nlm.nih.gov/snp) of the NCBI was searched for any single nucleotide polymorphisms (SNP) in the BCL6 gene sequence. This database contains human single nucleotide variations, microsatellites, and small-scale insertions and deletions, along with publication, population frequency, molecular consequence, and genomic and RefSeq mapping information for both common variations and clinical mutations. The obtained SNPs were filtered for missense mutations, which would lead to amino acid changes within the protein sequence. The population frequency of missense SNPs was also evaluated for the positions, which are involved in the interaction between the complexes of BCL6/BI and BCL6/DB. The clinical significance of these variations was analyzed within the ClinVar (https://www.ncbi.nlm.nih.gov/clinvar/) database of the NCBI. This database aggregates information about genomic variation and its relationship to human health.

### Analyzing the possible off-targets of BI-3802 and DB11611

The sequence of the BTB domain of BCL6 was used to perform a protein BLAST search (https://blast.ncbi.nlm.nih.gov/Blast.cgi). The search was limited to the PDB as the search database and the *Homo sapiens* as the target organism. These settings would result in similar human proteins with resolved 3D structures. The search algorithm was set to PSI-BLAST (Position-Specific Iterated BLAST). This algorithm allows the user to build a PSSM (position-specific scoring matrix) using the results of the first BLASTP run. This tool provides a means of detecting distant relationships between proteins. The search was iterated (10 iterations) until no new sequences were added to the search results. The top five non-BCL6 proteins were selected to analyze the possible binding with BI-3802 and DB11611.

### Molecular docking between off-target candidates, BI-3802, and DB11611

The structures of the top five selected proteins were obtained from PDB. The protein structures were docked against BI-3802 and DB11611 (Lifitegrast) using Autodock Tools software. The protein and ligand structures were prepared as PDBQT files. The grid box size was set to coordinates that include the whole proteins. The van der Waals and electrostatic terms were calculated using set- and distance-dependent dielectric functions of the AutoDock Vina software. The binding affinity of the BI-3802 and Lifitegrast was also calculated against the selected protein structures. The binding energy would be a good indicator of the stability of ligand and protein interaction.

### Passive membrane permeability

The PerMM web server (https://permm.phar.umich.edu/) is a computational tool for the theoretical evaluation of the passive permeability of molecules through the lipid bilayer. The PerMM method determines the permeability coefficients of various compounds across various membranes and calculates energy profiles along the bilayer and membrane binding affinity. The 3D structures of the BI-3802 and Lifitegrast were used as input files to calculate the passive membrane permeability.

## Supplementary Information

Supplementary Information 1.

Supplementary Information 2.

## Data Availability

The datasets analyzed during the current study are available in the Uniprot repository (https://www.uniprot.org/, accession number: P41182), RCSB PDB repository (https://www.rcsb.org/, accession number: 5MW2 and 6XMX), Binding Database repository (https://www.bindingdb.org/rwd/bind/index.jsp)^51^, DrugBank repository (https://www.drugbank.ca/, approved drugs)^52^, SNP repository (https://www.ncbi.nlm.nih.gov/snp), and ClinVar repository (https://www.ncbi.nlm.nih.gov/clinvar/).
