# Supplementary figures and images for: Impact of SNPs, off-targets, and passive permeability on efficacy of BCL6 degrading drugs assigned by virtual screening and 3D-QSAR approach
Source: Sci Rep. 2022 Dec 6;12:21091. doi: 10.1038/s41598-022-25587-3 (PMC9726907; doi:10.1038/s41598-022-25587-3)

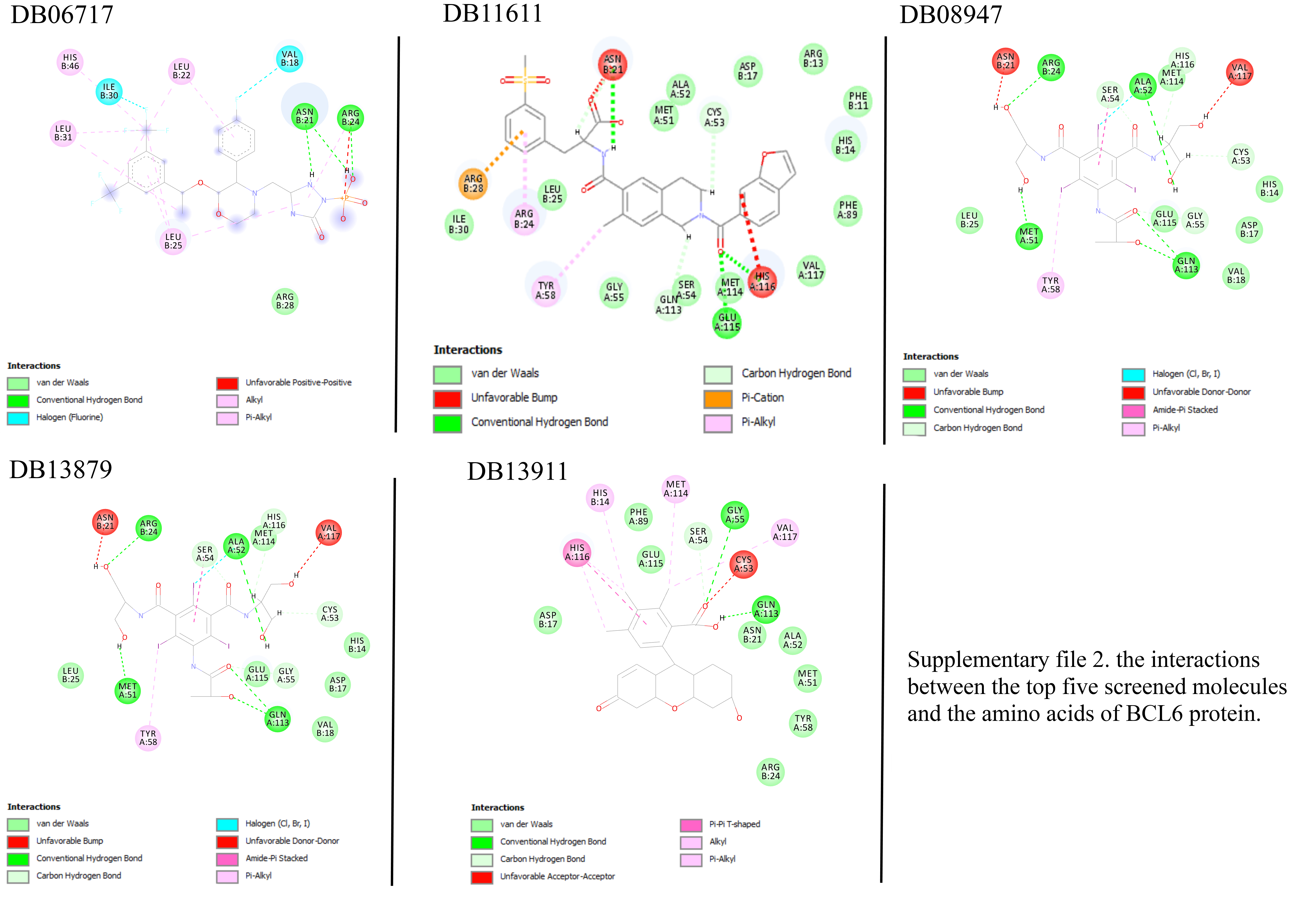

Supplement: Supplementary file 2 — Supplementary Information 2. [file 41598_2022_25587_MOESM2_ESM.tiff]
